# Supplementary material for: Population genetic structure and historical demography of the population of forest elephants in Côte d’Ivoire
Source: PLoS One. 2024 Aug 26;19(8):e0300468. doi: 10.1371/journal.pone.0300468 (PMC11346955; doi:10.1371/journal.pone.0300468)
Supplement: Attachment — Submitted filename: Responses to reviewers_gs_27janvier2024_2.docx [file pone.0300468.s002.docx]

**Responses to reviewers**

**Reviewer #1:**

This manuscript represents a high contribution towards the *Loxodonta cyclotis* genetic population and conservation. However, I have noticed some crucial issues that need to be addressed:

1) The text requires reorganization. For example, the methodology should clearly state how the sequences were curated leaving only 101 sequences and avoid re-presenting information already presented in the result section in the discussion section.

1) Answer

Of the 137 samples with a positive simplex PCR, 80.30 % (110 out of 137) were successfully sequenced. As part of this study, 47, 41 and 13 good quality sequences of the mitochondrial DNA control region (CSB1) obtained from dung samples respectively collected in the Bossématié (FCB), Dassioko (FCD) and Port-Gauthier (FCPG) Forest Reserves. These 130 sequences were taken into account for different analyses.

2) The utilization of a highly variable sequence to infer species diversity and population historical demography represents a weakness in this study. The authors should, at the very least, acknowledge their awareness of this limitation. In comparison, all other studies used for reference employed at least two genetic markers, including less variable regions. If the authors followed a similar approach, they should emphasize it in the manuscript.

2) Answer

The genetic profile established in this study is based on mitochondrial DNA (mtDNA) control region, which is only transmitted maternally. This non-coding genetic marker commonly used in conservation genetic studies to examine genetic structure at the population level because it is considered to be the most rapidly evolving region of the mitochondrial genome (Baker and Marshall 1997; Ruokonen 2002). This locus is broadly considered to be suitable for examining intraspecific relationships among closely related groups of individuals (Sinclair and Pe´rez-Losada 2005). However, the use of mtDNA control region presents some limitations resulting from the fact that the analysis of mtDNA corresponds to the study of a single locus. This means that the total dependence on mtDNA as a marker in the study of the natural history of populations will give only a small part of that history (Zhang & Hewitt 2003). Indeed, the high evolutionary rate that has made the control region an attractive marker for biologists may be masking the true relationships between populations due to high haplotype diversity as well as homoplasy. Hence, the use of an alternative marker with a slower evolutionary rate may be more suitable than the control region to reveal the population structure of forest elephant.

3) I noticed that the supplementary material mentioned in line 326 is missing, as well as the GenBank accession numbers of the sequences they obtained.

3) Answer

The GenBank accession numbers of the sequences obtained have been included as suggested by the reviewer. The additional material mentioned is available from line 521 to 523.

**Data Availability Statement:** Sequence data are available in GenBank <https://www.ncbi.nlm.nih.gov/nuccore/MZ485910.1,MZ485851.1,MZ485850.1>,

MZ485849.1,MZ485848.1,MZ485847.1,MZ485846.1,MZ485845.1,MZ485844.1,MZ485843.1,MZ485842.1,MZ485841.1,MZ485840.1,MZ485839.1,MZ485838.1,MZ485834.1,MZ485833.1,KJ557424.1,MZ485831.1,MZ485830.1,MZ485829.1,MZ485828.1,MZ485827.1,MZ485826.1,MZ485825.1,MZ485824.1,MZ485823.1,MZ485822.1,MZ485821.1,MZ485820.1,MZ485819.1,MZ485818.1,MZ485817.1,MZ485816.1,MZ485815.1,MZ485814.1,MZ485813.1,MZ485812.1,MZ485811.1,MZ485810.1,MZ485809.1,MZ485852.1,MZ485909.1,MZ485903.1,MZ485900.1,MZ485899.1,MZ485898.1,MZ485896.1,MZ485895.1,MZ485894.1,MZ485892.1,MZ485891.1,MZ485890.1,MZ485889.1,MZ485881.1,MZ485880.1,MZ485879.1,MZ485878.1,MZ485877.1,MZ485871.1,MZ485870.1,MZ485869.1,MZ485866.1,MZ485865.1,MZ485864.1,MZ485863.1,MZ485862.1,MZ485861.1,MZ485859.1,MZ485858.1,MZ485856.1,JQ438209.1,MZ485905.1,MZ485904.1,MZ485887.1,MZ485886.1,MZ485885.1,MZ485882.1,MZ485873.1,MZ485872.1,MZ485836.1,MZ485832.1,MZ485888.1,MZ485883.1,MZ485908.1,MZ485907.1,MZ485906.1,MZ485902.1,MZ485876.1,MZ485874.1,MZ485860.1,MZ485837.1,MZ485835.1,JQ438745.1,MZ485893.1,MZ485884.1,MZ485901.1,MZ485897.1,MZ485868.1,MZ485867.1

List the number of samples collected in the Genbank database used in this study (Tables 3).

**Table 3** Accession numbers for samples included in this study.

| Species | Sample origin | Accession number | Citation |
| --- | --- | --- | --- |
| *Loxodonta cyclotis* | Côte d’Ivoire | KJ557424.1 | [71] |
|  | Gabon | KJ557423 | [71] |
|  | Sierra Léone | JN673264 | [70] |
|  | République centrafricaine | JN673263 | [70] |
| *Loxodonta africana* | Savanne 1 | AB443879.1 | [80] |
|  | Savanne 2 | NC_000934.1 | [81] |
|  | Savanne 3 | DQ316069.1 | [82] |
| *Elephas maximus* | Asie 1 | DQ316068 | [73] |
|  | Asie 2 | NC_005129 | [73] |
|  | Asie 3 | EF588275 | [64] |
| *Mammuthus primigenius* | Mammouth | EU155210 | [83] |

4) Figure 4 and line 383: Based on this graphic, it is not possible to infer a correlation between genetic distance and geographical distance. While it is conceivable that this genetic marker should exhibit an isolation by distance pattern, the current graphic does not demonstrate such a relationship.

4) Answer

The Mantel test shows a strong and positive but not significant correlation, between genetic and geographical distances (Monte Carlo randomisation test, Rxy = 0.97; P = 0.37). Which does not suggest isolation by distance (IBD).

5) Phylogenetic relationships cannot be reliably inferred using only one variable genetic marker. I recommend refraining from drawing such conclusions throughout the text. As for Figure 5, it is evident that these individuals possess distinct haplotypes in comparison to other works, while also sharing similar haplotypes among themselves. However, no definitive conclusions about their phylogenetic relationships can be drawn from this data alone.

5) Answer

Figure 5 illustrates the relationships between the mtDNA control region sequences generated during this study and those of forest elephant (*Loxodonta cyclotis*), savanna elephant (*L. africana*), *Mammuthus primigenius*, and *Elephas maximus* retrieved from the National Center for Biotechnology Information (NCBI) database. The phylogenetic analysis of the subpopulations of the Bossématié, Dassioko, and Port Gauthier CFs shows that they appear monophyletic, with heterogeneous structuring (genetic variation) in a clade with the forest elephants selected in the NCBI da-tabase. Based on the sequence analysed, the phylogenetic study showed that forest elephants and savannah elephants form two different clades. This analysis detected that Asian elephants (E. maximus) and the Mammuthus primigenius clade form a clade in their own right, which differs from those of forest elephants and savannah elephants (Fig 5).

We are aware that Phylogenetic relationships cannot be reliably inferred using only one variable genetic marker. Particularly data from nonrecombining mitochondrial DNA (mtDNA), has important limitations that ares now widely recognized. Indeed, a mtDNA tree may be different from the population or species tree due to the effects of natural selection (see, for example, Weinreich & Rand 2000 and Bazin et al. 2006), introgression (Chan & Levin 2005; Melo-Ferreira et al. 2005) or the wide stochastic variance that characterizes a sample of gene trees collected from a set of populations or species (Hey & Machado 2003). Hence, no definitive conclusions about their phylogenetic relationships can be drawn from the obtained data alone.

**Reviewer #2:**

This is well-written manuscript and based on impressive empirical evidence and makes an original contribution. The abstract is adequately addressed. The Introduction is adequate. The literature review is concise updated covering the main relevant concept and empirical studies on different approaches on this field. In material methods sample collection, DNA extraction, PCR sequencing and data analysis is properly maintained as per this research work demand. The results are clearly presented with proper statistical methods and adequately addressed. The authors develop a unique theoretical framework and highlighted originality much more. The study may be assist to maintain elephant populations of Dassioko, Port Gauthier, Zoo of Abidjan and Bossematie. Therefore, the manuscript can be considered for the publication.

Good
